# Echocardiographic predictors of thrombus in left atrial appendage—The role of novel transthoracic parameters

**DOI:** 10.3389/fcvm.2022.1059111

**Published:** 2022-11-30

**Authors:** Damian Kaufmann, Elżbieta Wabich, Agnieszka Kapłon-Cieślicka, Monika Gawałko, Monika Budnik, Beata Uziębło-Życzkowska, Paweł Krzesiński, Katarzyna Starzyk, Beata Wożakowska-Kapłon, Maciej Wójcik, Robert Błaszczyk, Jarosław Hiczkiewicz, Jan Budzianowski, Katarzyna Mizia-Stec, Maciej T. Wybraniec, Katarzyna Kosmalska, Marcin Fijałkowski, Anna Szymańska, Mirosław Dłużniewski, Maciej Haberka, Michał Kucio, Błażej Michalski, Karolina Kupczyńska, Anna Tomaszuk-Kazberuk, Katarzyna Wilk-Śledziewska, Renata Wachnicka-Truty, Marek Koziński, Paweł Burchardt, Ludmiła Daniłowicz-Szymanowicz

**Affiliations:** ^1^Department of Cardiology and Electrotherapy, Medical University of Gdańsk, Gdańsk, Poland; ^2^“Club 30,” Polish Cardiac Society, Warsaw, Poland; ^3^1st Chair and Department of Cardiology, Medical University of Warsaw, Warsaw, Poland; ^4^West German Heart and Vascular Centre, Institute of Pharmacology, University of Duisburg-Essen, Essen, Germany; ^5^Department of Cardiology, Maastricht University Medical Centre, Cardiovascular Research Institute Maastricht, Maastricht, Netherlands; ^6^Department of Cardiology and Internal Diseases, Military Institute of Medicine, Warsaw, Poland; ^7^1st Clinic of Cardiology and Electrotherapy, Świętokrzyskie Cardiology Centre, Collegium Medicum Jan Kochanowski University, Kielce, Poland; ^8^Department of Cardiology, Medical University of Lublin, Lublin, Poland; ^9^Clinical Department of Cardiology, Nowa Sól Multidisciplinary Hospital, Nowa Sól, Poland; ^10^Collegium Medicum, Department of Interventional Cardiology and Cardiac Surgery, University of Zielona Góra, Zielona Góra, Poland; ^11^1st Department of Cardiology, School of Medicine in Katowice, Medical University of Silesia, Katowice, Poland; ^12^Department of Cardiology, St. Vincent Hospital, Gdynia, Poland; ^13^1st Department of Cardiology, Medical University of Gdańsk, Gdańsk, Poland; ^14^Department of Heart Diseases, Postgraduate Medical School, Katowice, Poland; ^15^Department of Cardiology, School of Health Sciences, Medical University of Silesia, Katowice, Poland; ^16^Department of Cardiology, Medical University of Łódź, Łódź, Poland; ^17^Department of Cardiology, Medical University of Białystok, Białystok, Poland; ^18^Department of Cardiology and Internal Medicine, Medical University of Gdańsk, Gdańsk, Poland; ^19^Department of Hypertension, Angiology, and Internal Medicine, Poznan University of Medical Sciences, Poznań, Poland

**Keywords:** transthoracic echocardiography, left atrial appendage thrombus, NOAC, echocardiographic indices, thromboembolic risk, predictors of left atrial thrombus

## Abstract

**Introduction:**

The left atrium appendage thrombus (LAAT) formation is a complex process. A CHA_2_DS_2_-VASc scale is an established tool for determining the thromboembolic risk and initiation of anticoagulation treatment in patients with atrial fibrillation or flutter (AF/AFL). We aimed to identify whether any transthoracic echocardiography (TTE) parameters could have an additional impact on LAAT detection.

**Methods:**

That is a sub-study of multicenter, prospective, observational study LATTEE (NCT03591627), which enrolled 3,109 consecutive patients with AF/AFL referred for transesophageal echocardiography (TEE) before cardioversion or ablation.

**Results:**

LAAT was diagnosed in 8.0% of patients. The univariate logistic regression analysis [based on pre-specified in the receiver operating characteristic (ROC) analysis cut-off values with AUC ≥ 0.7] identified left ventricular ejection fraction (LVEF) ≤ 48% and novel TTE parameters i.e., the ratios of LVEF and left atrial diameter (LAD) ≤ 1.1 (AUC 0.75; OR 5.64; 95% CI 4.03–7.9; *p* < 0.001), LVEF to left atrial area (LAA) ≤ 1.7 (AUC 0.75; OR 5.64; 95% CI 4.02–7.9; *p* < 0.001), and LVEF to indexed left atrial volume (LAVI) ≤ 1.1 (AUC 0.75, OR 6.77; 95% CI 4.25–10.8; *p* < 0.001) as significant predictors of LAAT. In a multivariate logistic regression analysis, LVEF/LAVI and LVEF/LAA maintained statistical significance. Calculating the accuracy of the abovementioned ratios according to the CHA_2_DS_2_-VASc scale values revealed their highest predictive power for LAAT in a setting with low thromboembolic risk.

**Conclusion:**

Novel TTE indices could help identify patients with increased probability of the LAAT, with particular applicability for patients at low thromboembolic risk.

## Introduction

Atrial fibrillation and flutter (AF/AFL) are the most common sustained cardiac arrhythmias in adults (1, 2), with thromboembolic complications as the main reason for morbidity and mortality (3). The CHA_2_DS_2_-VASc scale is an established clinical tool which is recommended for determining the thromboembolic risk and anticoagulation treatment indications in AF/AFL patients (4). However, thrombus formation is a complex process, which involves many hemorheological, tissue and humoral factors; hence the mere assessment of the thrombus mass formation based only on the abovementioned scale could be insufficient (5). Therefore, it could be reasonable to relate the CHA_2_DS_2_-VASc scale to some morphological parameters, which could have a possible impact on thrombus development, and echocardiography could be a valuable tool in this issue. Transesophageal echocardiography (TEE) is regarded as the gold standard in detecting the left atrial (LA) appendage thrombus (LAAT) before cardioversion or ablation procedure (6, 7). However, in certain situations, its performance is hampered or even not possible, for instance, due to logistical difficulties related to restricting access to the TEE in small district hospitals, as well as in certain situations, such as the COVID-19 pandemic, in which the implementation of the study was limited. Therefore, it seems reasonable to verify whether any routinely assessed transthoracic echocardiography (TTE) parameters could help identify patients with a high probability of LAAT, which could allow clinicians to avoid unnecessary diagnostics and influence the appropriate management of a patient.

Many studies have focused so far on the search for echocardiographic parameters that predict the risk of LAAT (8–11), revealing LA enlargement [both diameter (LAD), surface area (LAA), indexed volume (LAVI)], and decreased left ventricular ejection fraction (LVEF) as the most associated with thrombus formation. However, the predictive power of these conventional variables is insufficient (8, 9). Therefore, we hypothesized that perhaps parameters determining the size, area, and volume of the atrium, in combination with other echocardiographic parameters such as LVEF, may prove valuable as a marker of increased risk of LAAT formation in real-world AF/AFL patients referred for TEE before electrical cardioversion or catheter ablation in the era of modern anticoagulation.

## Materials and methods

### Study population

The study is a sub-analysis of the real-world Left Atrial Thrombus on Transesophageal Echocardiography (LATTEE) registry (NCT03591627), which evaluated the determinants of LAAT depending on echocardiographic and clinical parameters in patients with AF/AFL referred for electrical cardioversion or catheter ablation. Exact details on the study rationale and design have been published previously (12), while the primary data concerning the prevalence of a thrombus depending on anticoagulation strategy were further precisely delineated (13). In sum, the LATTEE was a prospective, observational study enrolling consecutive patients with AF/AFL admitted to 13 cardiology departments between November 2018 and May 2020 in whom TEE was performed before direct current cardioversion or catheter ablation. Diagnosis of AF/AFL was based on previous European Society of Cardiology Guidelines on managing AF by attending physicians (14). Regarding non-emergency electrical cardioversion for AF/AFL, four centers performed TEE routinely in all patients, and nine centers performed TEE only in those patients who were suspected of ineffective antithrombotic therapy within the last 3 weeks. The study was conducted according to clinical practice guidelines and the Declaration of Helsinki. The Ethics Committee approved the study of the Medical University of Warsaw (AKBE/113/2018), which waived the requirement of obtaining informed consent from the patients.

### Data collection and study endpoint

Data were gathered prospectively and included precise demographics, medical history, comorbidities, CHA_2_DS_2_-VASc score calculation, pharmacotherapy, and results of routine laboratory blood tests. Chronic oral anticoagulation (OAC) was defined as OAC treatment for at least 3 weeks before the procedure. In all patient’s obligatory transoesophageal echocardiography (TOE) parameters such as presence and location of LAAT, presence of spontaneous echocardiographic contrast, as well as LAA outflow velocity (LAAV) were obtained. TTE study was conducted in the vast majority of participants and involved gathering data regarding: LVEF, LAD, LAA, left atrial volume (LAV) and LAVI (calculated as a ratio of left atrial volume to body surface area). Trained echocardiographers performed all examinations as it was defined in the primary protocol (12). Additionally, the novel parameters (ratios of LVEF and LA parameters: LVEF/LAD, LVEF/LAA, and LVEF/LAVI) were investigated. Both TTE and TOE parameters were analyzed and interpreted locally. The primary endpoint of the study was the presence of LAAT.

### Statistical analysis

Continuous data were presented as the median (25th–75th percentiles), categorical as a number (n) and percentage (%). Differences between LAAT+ and LAAT- groups were calculated with the Mann-Whitney *U*-test and the qualitative data with the χ^2^ or Yates χ^2^ test. The accuracy of pre-specified cut-off values for analyzed parameters and their association as potential predictors of the study endpoint was determined by area (AUC) under the receiver operating characteristic (ROC) curve. Only AUC values ≥ 0.7 were considered for further analysis (15). For comparison of unpaired ROC curves Venkatraman’s test was utilized. The association between the analyzed parameters (differed between LAAT+ and LAAT- groups) and the endpoint was assessed using univariable logistic regression analysis with cut-off values pre-specified in ROC analysis. Multivariable analysis was applied to continuous data (dichotomized according to the cut-off values identified in ROC analyses) and categorical data associated with the endpoint in the univariable regression analysis (*p* ≤ 0.05). The set of variables accepted for the model was determined by the backward elimination method from the set of all statistically significant predictors. The statistical analysis was conducted with an R 4.0.5 environment (R Core Team, Vienna, Austria).

## Results

### Study population

A total of 3,109 patients who met the inclusion criteria were enrolled in the LATTEE registry. Altogether, nearly 9 out of 10 were on OACs. Prevalence of LAAT was 8.0% (7.3% on chronic OAC vs. 15% without OAC; *p* < 0.001) and it was doubled in patients on vitamin K antagonist (VKA) compared to patients on non-VKA-OACs (NOACs) (13 vs. 6.0%; *p* < 0.01). Patients with LAAT were older and more often had chronic AF and comorbidities, resulting in a higher CHA_2_DS_2_-VASc score, as shown in Table 1. All clinical parameters of the study population were presented in previous work (13).

**TABLE 1:** Comparison of the clinical characteristics between patients with (LAAT+) and without LAAT (LAAT).

Variable	LAAT- (*n* = 2,859)	LAAT+ (*n* = 250)	*p* ^a^
**Demographics**			
Age (years)	67 [59–73]	72 [64–78]	<0.001
**AF/AFL type**			
AF/AFL paroxysmal	1,247 (44%)	33 (13%)	<0.001
AF/AFL persistent	1,365 (48%)	183 (73%)	<0.001
AF/AFL long-standing persistent	237 (8%)	34 (14%)	0.007
AF chronic	109 (4%)	20 (8%)	0.004
**Comorbidities**			
Heart failure	1,165 (41%)	171 (69%)	<0.001
Heart failure with reduced LVEF	380 (13%)	96 (39%)	<0.001
Hypertension	2,171 (76%)	195 (79%)	0.393
Diabetes mellitus	683 (24%)	91 (37%)	<0.001
Previous stroke	206 (7.2%)	29 (12%)	0.017
TIA	75 (3%)	15 (6%)	0.005
Previous ischemic stroke/TIA/systemic embolism	278 (9.7%)	35 (14%)	0.040
Previous hemorrhagic stroke	14 (0.5%)	3 (1.2%)	0.148
Vascular disease	949 (33%)	118 (47%)	<0.001
Myocardial infarction	372 (13%)	59 (28%)	<0.001
Coronary artery disease	811 (29%)	94 (38%)	0.002
Peripheral artery disease	149 (5%)	26 (10%)	<0.001
Moderate to severe mitral stenosis	12 (0.4%)	5 (2%)	0.009
Moderate to severe mitral regurgitation	442 (15%)	81 (32%)	<0.001
Moderate to severe aortic stenosis	47 (1.6%)	15 (6%)	<0.001
CIED	341 (12%)	57 (23%)	<0.001
eGFR < 50 (mL/min)	82 [64–103]	74 [51–93]	<0.001
Previous bleeding	114 (4.0%)	17 (6.9%)	0.05
Anemia	431 (16%)	53 (23%)	<0.01
Labile INR	50 (2%)	23 (9%)	<0.001
Smoking	902 (33%)	109 (46%)	<0.001
Alcohol	106 (4%)	23 (9%)	<0.001
**Thromboembolic risk and indications to chronic OAC**			
CHA_2_DS_2_-VASc score	3 [2–4]	4 [3–5]	0.010
**Antithrombotic therapy**			
Chronic OAC therapy	2,553 (89%)	200 (80%)	<0.001

^a^*p*-value refers for the differences between LAAT (+) and LAAT (-) groups.

AF, atrial fibrillation; AFL, atrial fibrillation; CIED, cardiac implanted electrical device; eGFR, estimated glomerular filtration rate; INR, international normalized ratio; LAAT, left atrial appendage thrombus; MS, mitral stenosis; OAC, an oral anticoagulant. TIA, transient ischemic attack.

### Echocardiographic parameters

TTE data were obtained for 2,599 (84%) study participants, and Table 2 presents the results. LAAT+ patients had lower LVEF and greater LAD, LAA, LAV, and LAVI values. The compared groups differed significantly in terms of the echocardiographic indices, i.e., LAAT+ in comparison to LAAT- patients had a lower ratio of LVEF to LA indices: LVEF/LAD 0.9 vs. 1.2 (*p* < 0.001), LVEF/LAA 1.4 vs. 2.1 (*p* < 0.001), and LVEF/LAVI 0.7 vs. 1.2 respectively (*p* < 0.001), as shown in Table 2.

**TABLE 2:** Comparison of LVEF, LA parameters and ratios in LAAT+ and LAAT- patients.

Variable	LAAT- (*n* = 2,859)	LAAT+ (*n* = 250)	*p* ^a^
LVEF (%)	55 [45–60]	40 [30–51]	<0.001
LAD (mm)	45 [41–49]	47 [45–51]	<0.001
LAA (cm^2^)	26 [22–30]	28 [24.8–33]	<0.001
LAV (ml)	85 [69–109]	97 [76–123]	<0.001
LAVI (mL/m^2^)	44 [35–55]	52 [42.9–63]	<0.001
LVEF/LAD ratio	1.2 [0.98–1.4]	0.9 [0.62–1.09]	<0.001
LVEF/LAA ratio	2.1 [1.6–2.51]	1.4 [0.97–1.83]	<0.001
LVEF/LAVI ratio	1.2 [0.88–1.57]	0.7 [0.52–1.05]	<0.001

^a^*p*-values refer for the differences between LAAT+ and LAAT- groups.

LAA, left atrial area; LAD, left atrial diameter; LAAT, left atrial appendage thrombus; LAV, left atrial volume; LAVI, left atrial volume index; LVEF, left ventricular ejection fraction.

### Significant predictors of left atrial thrombus

Table 3 presents the results of ROC analysis with pre-specified cut-off values for LAAT prediction. The LA parameters alone did not have adequate predictive power (AUC lower than < 0.7), whereas ratios of LVEF with LA parameters significantly improved the level of LAAT prediction with high specificity and positive predictive value.

**TABLE 3:** Accuracy of the pre-specified cut-off values for analyzed parameters as the predictors of LAAT.

Parameter	AUC	Characteristics (%)		Predictive value (%)	
			
		Sensitivity	Specificity	Positive	Negative
Age ≥ 72 years	0.61	74	46	94	13
LVEF ≤ 48%	0.74	71	65	96	17
LAD ≥ 45 mm	0.63	53	67	95	12
LAA ≥ 26 cm^2^	0.62	53	66	94	12
LAV ≥ 89 mL	0.59	55	59	93	11
LAVI ≥ 51 mL/m^2^	0.64	68	54	94	14
LVEF/LAD ratio ≤ 1.1	0.75	62	79	97	16
LVEF/LAA ratio ≤ 1.7	0.75	71	70	96	18
LVEF/LAVI ratio ≤ 1.1	0.75	56	84	97	15

AUC, an area under the curve; CI, confidence interval; LAA, left atrial area; LAD, left atrial diameter; LAAT, left atrial appendage thrombus; LAV, left atrial volume; LAVI, left atrial volume index; LVEF, left ventricular ejection fraction.

**FIGURE 1:**
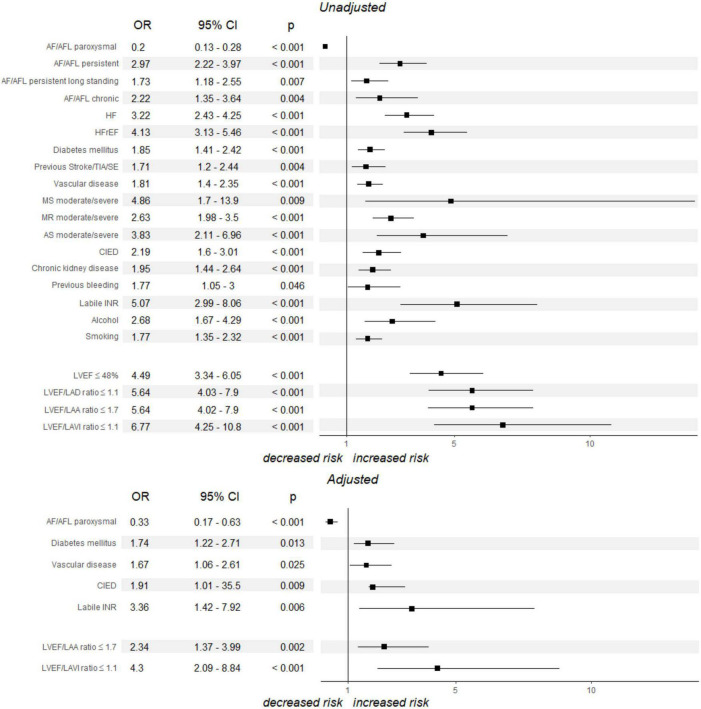
Univariate and multivariate logistic regression analysis models estimating the likelihood of LAAT. *p*-values refer for the differences between LAAT+ and LAAT- groups. AF, atrial fibrillation; AFL, atrial flutter; AS, aortic stenosis; CI, confidence interval; CIED, cardiac implanted electrical device; HF, heart failure; HFrEF, heart failure with reduced left ventricular ejection fraction; INR, international normalized ratio; LAA, left atrial area; LAAT, left atrial appendage thrombus; LAD, left atrial diameter; LAVI, left atrial volume index; LVEF, left ventricular ejection fraction; MR, mitral regurgitation; MS, mitral stenosis; OR, odds ratio; SE, systemic embolism; TIA, transient ischemic attack.

The univariate logistic regression analysis (based on pre-specified in the ROC analysis cut-off values with AUC ≥ 0.7) revealed a considerable number of clinical parameters, as well as LVEF, LVEF/LAD, LVEF/LAA and LVEF/LAVI ratios as the significant predictors for LAAT. These data are presented in Figure 1. C-Statistics analyses showed that the accuracy power of new echocardiographic indices (LVEF/LAD, LVEF/LAA, LVEF/LAVI ratios) differed significantly from conventional parameters (LAD, LAA, LAVI—in all combinations *p* < 0.05) but not for LVEF (*p* > 0.05). In a multivariate logistic regression analysis, which included all parameters which proved to be statistically significant in the univariate test (with AUC ≥ 0.7 for continuous variables from Table 3), only a few clinical parameters, as well as LVEF/LAVI and LVEF/LAA ratio maintained its statistical significance, as shown in Figure 1.

### Significant predictors of left atrial appendage thrombus in subpopulation of patients with heart failure

Among the entire study population, 43% of the patients, i.e., 1,336, were diagnosed with heart failure (HF). Of the HF types, the most common was HF with preserved ejection fraction (HFpEF), then reduced ejection fraction (HFrEF) and mid-range ejection fraction (HFmEF), 38, 35, and 27%, respectively. Most HF patients had symptoms consistent with NYHA I-II (72%).

The results of logistic regression analysis and ROC with specific cut-off values for LAAT prediction in patients with HF subtypes are presented in Table 4. In each of the HF subtypes tested, AUC and OR values were lower than those obtained for the entire study population. The new echocardiographic indices differed in statistical power depending on the HF subtype, and more precisely, they had highest prediction for LAAT formation in patients with HFpEF, where they obtained acceptable values for LVEF/LAA ≤ 1.8 [AUC 0.7, OR 4.1, 95% CI (1.9–9), *p* = 0.001) and LVEF/LAVI ≤ 1.1 [0.71, OR 4.4, 95% CI (1.7–11.6), *p* = 0.003].

**TABLE 4:** Univariate regression analysis and ROC study results of novel echocardiographic parameters in subpopulation of patients with HF.

Parameter	AUC	OR 95%	*p* ^a^
**HF with reduced EF**			
LVEF/LAA ≤ 1.0	0.59	2 [1.2–3.4]	0.01
LVEF/LAVI ≤ 0.6	0.56	2.1 [1.1–4.1]	0.023
**HF with mid-range EF**			
LVEF/LAA ≤ 1.5	0.68	2.2 [1.1–4.6]	0.033
LVEF/LAVI ≤ 0.9	0.60	3.8 [3.6–9.2]	0.002
**HF with preserved EF**			
LVEF/LAA ≤ 1.8	0.70	4.1 [1.9–9]	0.001
LVEF/LAVI ≤ 1.1	0.71	4.4 [1.7–11.6]	0.003
**HF with mid-range EF and HF with reduced EF**			
LVEF/LAA ≤ 2	0.67	4.3 [2.1–8.9]	0.001
LVEF/LAVI ≤ 1	0.72	4.5 [2.3–8.5]	0.001

^a^*p*-values refer for the differences between LAAT (+) and LAAT (-) groups.

AUC, an area under the curve; EF, ejection fraction; HF, heart failure; LAA, left atrial area; LAVI, left atrial volume index; LVEF, left ventricular ejection fraction; OR, odds ratio.

### Accuracy of transthoracic echocardiographic indices for left atrium appendage thrombus detection according to CHA_2_DS_2_-VASc score values

Based on the statistical significance of novel echocardiographic ratios, we determined their odds ratio for LAAT prediction in different CHA_2_DS_2_-VASc groups. For this purpose, we divided patients into three subgroups accordingly to (A) 0–1, (B) 2–3, and (C) 4 and more points on the CHA_2_DS_2_-VASc score. In ROC analysis, the appropriate cut-off values for LAAT prediction were determined, as shown in Table 5. The obtained data show that the discussed indices were characterized by better accuracy and predictive power than conventional parameters, and that the LVEF/LAA index predicts the formation of LAAT with the highest statistical power.

**TABLE 5:** Accuracy of echocardiographic indices in LAAT prediction according to CHA_2_DS_2_-VASc score values.

Parameters	AUC	Characteristics (%)		Predictive value (%)		OR 95%	*p* ^a^
		Sensitivity	Specificity	Positive	Negative		
**Subgroup A (CHA_2_DS_2_-VASc 0–1 point)**							
LVEF/LAA ≤ 1.5	0.92	89	89	100	17	29 [5.9–145]	<0.001
LVEF/LAVI ≤ 0.7	0.78	92	60	98	21	18 [4.7–68]	<0.001
LVEF ≤ 48%	0.72	85	62	99	9	9 [2.8–25]	<0.001
LAD ≥ 41 mm	0.74	37	100	100	4	15 [0.9–264]	0.003
LAA ≥ 26 cm^2^	0.79	62	100	100	6	29 [1.7–511]	<0.001
**Subgroup B (CHA_2_DS_2_-VASc 2–3 points)**							
LVEF/LAA ≤ 1.5	0.77	78	72	97	22	9 [5–16.2]	<0.001
LVEF/LAVI ≤ 0.9	0.75	77	67	96	21	6.6 [3.5–12.2]	<0.001
LVEF ≤ 47%	0.76	74	68	96	19	5.9 [3.7–10]	<0.001
LAD ≥ 45 mm	0.67	53	74	96	12	3.2 [1.87–5.2]	<0.001
LAA ≥ 29 cm^2^	0.67	69	58	95	14	3.1 [1.8–5.1]	<0.001
**Subgroup C (CHA_2_DS_2_-VASc 4 and more points)**							
LVEF/LAA ≤ 2.0	0.70	43	87	96	17	4.3 [2.5–7.4]	<0.001
LVEF/LAVI ≤ 0.9	0.69	70	64	94	20	3.7 [2.3–6.1]	<0.001
LVEF ≤ 55%	0.70	38	92	98	15	7.7 [3.7–14.3]	0.001
LAD ≥ 44 mm	0.55	39	72	92	13	1.7 [1.1–2.5]	0.019
LAA ≥ 29 cm^2^	0.55	29	82	92	13	1.8 [1.1–3.1]	0.02

^a^*p*-values refer for the differences between LAAT (+) and LAAT (-) groups.

AUC, an area under the curve; LAA, left atrial area; LAD, left atrial diameter; LAAT, left atrial thrombus; LVEF, left ventricular ejection fraction; OR, odds ratio.

## Discussion

The major finding in this prospective, observational study is that LAAT formation was strongly associated with echocardiographic parameters, additionally to well-known clinical variables. We determined that simple, routinely examined echocardiographic parameters presented as the novel indices, including LVEF and LA parameters seem to be accurate predictors of LAAT presence, mainly according to different CHA_2_DS_2_-VASc score groups with peculiar applicability for patients with relatively lower thromboembolic risk.

To date, several risk stratification methods utilizing clinical parameters have been developed to help pinpoint patients with AF/AFL who are at high risk for thromboembolic complications, among which the most recognized is the CHA_2_DS_2_-VASc score (16). Nonetheless, some other investigators had a differing viewpoint on this issue (17, 18). The role of data derived from the TTE study as a marker of LAAT formation has been studied extensively over the last decades (10, 19–22). For example, in the study of Scherr et al., which enrolled 585 patients referred for catheter ablation of AF, LAD ≥ 45 mm and a CHADS_2_ score ≥ 2 proved to be significant predictors of LA thrombus in multivariate regression analysis (10). Our data are in line with those observations. Moreover, the capacity for predicting LAAT by combining LA area and volume parameters and LVEF seems stronger than using any single echocardiographic parameter. In our study, we proposed some novel echocardiographic indices, easy to obtain from the routinely checked parameters, which could have an additional impact on LAAT detection. Of the TTE indices, the LVEF/LAD with a cut-off value of ≤ 1.1, LVEF/LAA ratio ≤ 1.7 and LVEF/LAVI ≤ 1.1 had the highest predictive accuracy (AUC ≥ 0.7) predictive power and statistical significance in the univariate logistic regression analysis. Importantly, in the multivariate logistic regression analysis, LVEF/LAVI and LVEF/LAA maintained statistical significance.

For better prediction of LAAT, models combining clinical and echocardiographic parameters have been proposed (17, 19–24). For example, Van Chien et al., in their study of 144 anticoagulant-naïve patients, proposed models that combined CHA_2_DS_2_-VASc score with LA volume index and LA longitudinal strain (17). In another study conducted by Ayirala et al. on 334 patients who received VKA or VKA and heparin, the authors showed that patients with CHADS_2_ score of ≤ 1 a normal LAVI in combination with normal LVEF are a robust negative predictor of LAA thrombus formation (19). Our results are under data from the literature; indeed, the calculation of LVEF/LAVI and LVEF/LAA ratio in different CHA_2_DS_2_-VASc score groups had a significant association with LAAT. Notably, the highest OR for LAAT prediction of presented echocardiographic indexes is for patients with low thromboembolic risk (Table 5). For example, LVEF/LAA index ≤ 1.5 in low-risk patients (with 0 or 1 points in CHA_2_DS_2_-VASc score) was characterized by an OR 29, CI 5.87–145.52 with an excellent AUC equal to 0.92. Similarly, the positive predictive value of the pre-specified cut-offs was higher for patients with a lower CHA_2_DS_2_-VASc score. That could be of great clinical value, helping clinicians identify patients with a high likelihood of LAAT, regardless of a low CHA_2_DS_2_-VASc score.

HF patients constitute a special population within atrial fibrillation patients, and their increasing coexistence is associated with significantly elevated in-hospital mortality (25). The occurrence of AF in patients with HF may lead to clinical disease progression and increases mortality, on the other hand, presence of HF in AF patients interfere with preservation of sinus rhythm through atrial remodeling, increases the number of strokes and mortality (26, 27). Despite the fact that congestive HF is a part of CHA_2_DS_2_-VASc score whether every HF subtype generates the same risk of LAAT formation is still in question (28, 29). In a recently published work, also based on data from the LATTEE registry Wybraniec et al. examined a population of 1,336 patients with HF and showed that the diagnosis of HFrEF, but neither HFmrEF nor HFpEF, confers a considerable risk of LAT formation (30). In our study we evaluated the usefulness of the new echocardiographic parameters i.e., LVEF/LAA and LVEF/LAVI in all HF subtypes, however, the results are unsatisfactory and indicate the need to look for other LAT predictors in this group of patients.

Based on our results, it could be suggested that clinical risk scores should be combined with echocardiographic parameters to receive the most accurate data regarding LAAT formation. A significant advantage of our results boosts the fact that our research was based on a large, modernly anticoagulated group of patients, 82% of whom were on chronic NOAC. To the best of our knowledge, this is the first study that shows the usefulness of novel echocardiographic parameters in clinical presentation in identifying high-risk individuals of LAAT occurrence in the era of contemporary anticoagulation.

## Limitations of the study

Our study has some limitations. Firstly, the study was a registry and therefore has a limitation of its design. Secondly, despite the fact that we included a relatively large group of patients with AF/AFL, by inclusion criteria these were patients admitted for ablation or cardioversion procedures and therefore, the results cannot be extrapolated to the whole population of patients with AF/AFL. Thirdly, it is worth noting that echocardiographic study was performed at the discretion of attending physicians, and thus, data including TTE are missing for some patients. Moreover, a few promising parameters, such as LV stroke volume, LV end-systolic and end-diastolic volume as well as parameters of left ventricular diastolic dysfunction and peak atrial longitudinal strain that could identify patients at increased risk of LAAT, were not included in the methodology of that registry (31, 32). Additionally, the study did not investigate into the rate of ischemic stroke on follow-up, but only the presence of LAAT. Furthermore, TOE was performed routinely in most centers prior to direct current cardioversion and catheter ablation, however, some participating centers performed TOE only in subjects with suboptimal anticoagulation before the procedure or in those with doubts regarding adherence to NOAC and its effectiveness which might have led to some selection bias. Finally, study aimed to check which echocardiographic parameters can predict LAAT formation based on regular patients qualified to TEE in the everyday clinical practice hence we did not exclude a peculiar group of patients with “valvular AF” from the analysis.

## Conclusion

Simple, routinely examined echocardiographic parameters presented as the novel indices, including LVEF and LA parameters, seem to be accurate predictors of LAAT presence. Further use of those parameters could help predict LAAT in different CHA_2_DS_2_-VASc score groups with particular applicability for patients with low thromboembolic risk.

## Data availability statement

The raw data supporting the conclusions of this article will be made available by the authors, without undue reservation.

## Ethics statement

The studies involving human participants were reviewed and approved by the Ethics Committee of the Medical University of Warsaw. Written informed consent for participation was not required for this study in accordance with the national legislation and the institutional requirements.

## Author contributions

DK and LD-S: formal analysis, resources, writing—original draft preparation, visualization, data curation, and agreed to the published version of the manuscript. AK-C, MG, and MB: conceptualization, methodology, validation, investigation, data curation, writing—review and editing, project administration, and agreed to the published version of the manuscript. EW, BU-Ż, PK, KS, MCW, RB, JH, JB, KM-S, MTW, KTK, MF, AS, MD, MH, MCK, BM, KRK, AT-K, KW-Ś, RW-T, MRK, and PB: investigation, data curation, writing—review and editing, and agreed to the published version of the manuscript. LD-S: conceptualization, methodology, resources, writing—original draft preparation, visualization, supervision, data curation, and agreed to the published version of the manuscript. All authors contributed to the article and approved the submitted version.
